# Educational concussion module for professional footballers: from systematic development to feasibility and effect

**DOI:** 10.1136/bmjsem-2018-000490

**Published:** 2019-03-07

**Authors:** Vincent Gouttebarge, Charlotte Cowie, Edwin Goedhart, Simon P T Kemp, Gino M M J Kerkhoffs, Jon Patricios, Keith A Stokes

**Affiliations:** 1 World Players’ Union (FIFPro), Hoofddorp, Netherlands; 2 Amsterdam UMC, Univ of Amsterdam, Department of Orthopaedic Surgery, Amsterdam Movement Sciences, Meibergdreef 9, Amsterdam, Netherlands; 3 Amsterdam Collaboration on Health & Safety in Sports (ACHSS), AMC/VUmc IOC Research Center, Amsterdam, Netherlands; 4 Division of Exercise Science and Sports Medicine, University of Cape Town, Cape Town, South Africa; 5 The Football Association, London, UK; 6 Royal Netherlands Football Association (KNVB), FIFA Medical Center of Excellence, Zeist, Netherlands; 7 Rugby Football Union, Twickenham, UK; 8 School of Therapeutic Sciences, Faculty of Health Sciences, University of the Witwatersrand, Johannesburg, South Africa; 9 Department for Health, University of Bath, Bath, UK

**Keywords:** concussion, soccer, behaviour, education

## Abstract

**Objectives:**

To describe: (1) how we developed a concussion module and (2) whether the concussion module is feasible (in terms of relevance, added value and suitability) and enhances knowledge and changes attitude of professional footballers about concussion.

**Developing the concussion module:**

We developed the concussion module based on two structured and systematic processes. First, our needs assessment (questionnaire and interviews) in professional football (especially players) revealed that a 5–10 min concussion module was needed, ideally disseminated during club visits. Second, the objectives were defined (from published literature and by experts) as to disseminate essential information about what concussion is (definition), how to recognise it and the importance of removing a player with (suspected) concussion from the football field. We included an introductory video featuring a high-profile professional footballer and an animated educational component on defining concussion, recognising it and removing affected players from the field.

**Feasibility and effect:**

A quasiexperimental study (pretest post-test design) was conducted among 61 professional footballers. These players were asked to complete two questionnaires related to knowledge about and attitude towards concussion and feasibility of the module: one before and one after viewing the concussion module. Potential increase in knowledge and attitude was explored by comparing the pretest and post-test scores of the Rosenbaum Concussion Knowledge and Attitudes Survey with the non-parametric Wilcoxon signed-rank test (p<0.05). The mean knowledge score of the participants was stable between tests (Z=213; p=0.16), while mean attitude score increased significantly (Z=331; p=0.01). Nearly all participants (85%–100%) were positive about the relevance, added value, duration and form of the concussion module.

**Conclusion:**

The developed educational concussion module leads to better attitude of professional footballers towards concussion.

**What this study adds?**

- Following a structured and systematic approach, and relying especially on the needs and view of professional footballers, we developed an educational concussion module.
- The educational concussion module consists of an introductory video featuring by a high-profile professional footballer and an animated video focussing on the definition and recognition of concussion, and on the importance of removing a player with (suspected) concussion from the football field.
- Professional footballers were unanimously positive about the relevance, added value, form and duration of the educational concussion module.

## Introduction

Concussion is a complex pathophysiological process induced by biomechanical forces after a direct or transmitted blow to the head resulting in neurological impairment.^1^ In recent years, sports concussions have been increasingly under scrutiny, particularly because of their high incidence in collision sports and their potential long-term health consequences.^2–4^ Therefore, while continued efforts at improving its management are promoted, the prevention of concussions is being prioritised. For instance, in rugby, preactivation and coaching tackle technique have received greater emphasis in high-level youth rugby with the goal of decreasing the risk of injuries, including concussion, while the application of concussion management guidelines is monitored by the international and national organising bodies.^5–7^

In professional football, concussions are not very common with incidence rates ranging between 0.03 and 0.07 concussions per 1000 player hours in men’s European professional football (soccer).^8^ During the last five World Cups of the Fédération Internationale de Football Association (FIFA; 1998–2014), only 12 concussions were reported.^9^ However, the number of concussions in the 2014 World Cup in Brazil (n=5) and especially their on-field management caused a lot of discussion, emphasising how important it is to thoroughly educate managers, players and medical professionals about the appropriate management of concussion. Being the sole representative of professional footballers worldwide, the World Players’ Union (FIFPro) has initiated the development and implementation of an educational concussion module for professional footballers. This article describes (1) the systematic development of the concussion module and (2) whether the concussion module is feasible (in terms of relevance, added value and suitability) and contributes to enhancing the knowledge and attitude of professional footballers towards the injury.

## Development of the concussion module

In health promotion and sport research, interventions have been developed using structured and systematic processes such as the Intervention Mapping (IM) and the Knowledge Transfer Scheme (KTS).^10–12^ The development of the concussion module relied largely on the IM and KTS structured and systematic processes, following four subsequent steps: (1) needs assessment; (2) formulating its objectives; (3) selection of its content; (4) its development.^10–12^

### Step 1: needs assessment

The needs assessment was mainly conducted in order to assess the needs and support for the concussion module in professional football and also to explore its objectives and discuss the strategies for its implementation.

An electronic questionnaire (available in English, Dutch, French and Spanish) was designed and distributed between December 2016 and May 2017 by the World Players’ Union (FIFPro) to professional footballers and national professional footballers’ unions. Questions were related to (1) concussion policy and management, (2) potential improvements of concussion management, (3) needs about an educational concussion module and/or (4) the implementation of the concussion module. Participants were asked to give their informed consent (electronically) and to anonymously complete their questionnaire (FluidSurveys, Ottawa, Canada) within 2 weeks. Once completed (around 10 min was needed), the electronic questionnaires were saved automatically on a secured electronic server. A convenience sample of 306 participants (88% response rate) completed the questionnaire. Two out of three players (65%) who responded were in favour of education about concussion and its management, 90% of them mentioning that their representatives (players’ unions) were responsible for the dissemination of information during club visits. The large majority of the participants (91%) reported that the concussion module should last between 5 and 10 min. Other principal outcomes of the questionnaire are presented in box 1.

Box 1. **An educational concussion module: needs assessment in professional football**

**Current concussion management**

- 45% of the respondents mentioned that there was improvement needed in the current management of concussion in professional football.

**Return to play**

- 60% of the respondents mentioned that the decision about the return to sport of a player with (suspected) concussion should be taken by the medical team.
- 2% of the respondents mentioned that the decision about the return to sport of a player with (suspected) concussion should be taken by the player.
- 38% of the respondents mentioned that the decision about the return to sport of a player with (suspected) concussion should be taken by the medical team together with the player.

**Needs of information**

- 65% of the respondents mentioned that more and better information about concussion was needed for players.
- 50% of the respondents mentioned that more and better information about concussion was needed for coaches/managers.

**Responsibility**

- 89% of the respondents mentioned that the players’ representatives were responsible for the dissemination of more and better information about concussion.

**Concussion module**

- 55% of the respondents mentioned that an educational concussion module should last 5 min and 36% mentioned 10 min.
- 84% of the respondents mentioned that an educational concussion module should be digital of nature (film, animated video).
- 74% of the respondents mentioned that an educational concussion module could be implemented during the club visits of players’ representatives.
- 74% of the respondents mentioned that the collaboration between players’ representatives and football associations/federations should be favourable for the implementation of an educational concussion module.

Twelve semistructured face-to-face interviews (around 15 min) were held between May and August 2017 with six professional footballers and six national players’ associations from relevant sports in which concussion modules are already available (American Football, ice hockey, Rugby Union). Information collected through interviews confirmed the outcomes of the questionnaires and also revealed that the use of ambassadors (high profile players) might facilitate the implementation of an educational concussion module in professional football.

### Step 2 and step 3: objectives and contents of the concussion module

A review of the scientific literature was conducted using the electronic database MEDLINE (via PubMed; June 2017) in order to gather scientific evidence on the incidence, aetiology, prevention and management of concussions in sports, with a strong focus on professional football. An interdisciplinary meeting with an expert panel was held in order to explore the objectives and contents of the concussion module. Thirteen participants were selected with regard to their expertise and experience in concussion and/or professional football: four sports physicians, three retired professional footballers with histories of concussion, three representatives from national professional footballers’ unions, two communication experts and one academic professor. Led by an experienced academic, the discussion between experts was fed by the information gathered previously through the needs assessment and the scientific literature. The expert meeting took 6 hours and was held in November 2017.

The Fifth International Consensus Statement on Concussion in Sport (and related systematic review articles) informed discussion during the expert meeting.^1^ With regard to its intended duration (5 min), the experts selected the main objective for the educational concussion module, namely to disseminate essential information about the definition of concussion, recognition of concussion and the importance of removing a player with (suspected) concussion from the football field. As the concussion module needed to be used during the club visits of national professional footballers’ unions and available in English, French and Spanish (FIFPro’s official languages), the experts agreed to build the concussion module as follows: (1) an introductory video (between 1 and 1.5 min) featuring one to three ambassadors (native speaker in English, French and/or Spanish) being high profile professional footballers known by footballers worldwide and (2) an animated video focussing on the main information (definition, recognition, removal from the field), using voice-over (easy to translate into languages other than English, French and Spanish) and using recent video footage of clear concussive events in professional football.

### Step 4: development of the concussion module

**Figure 1:**
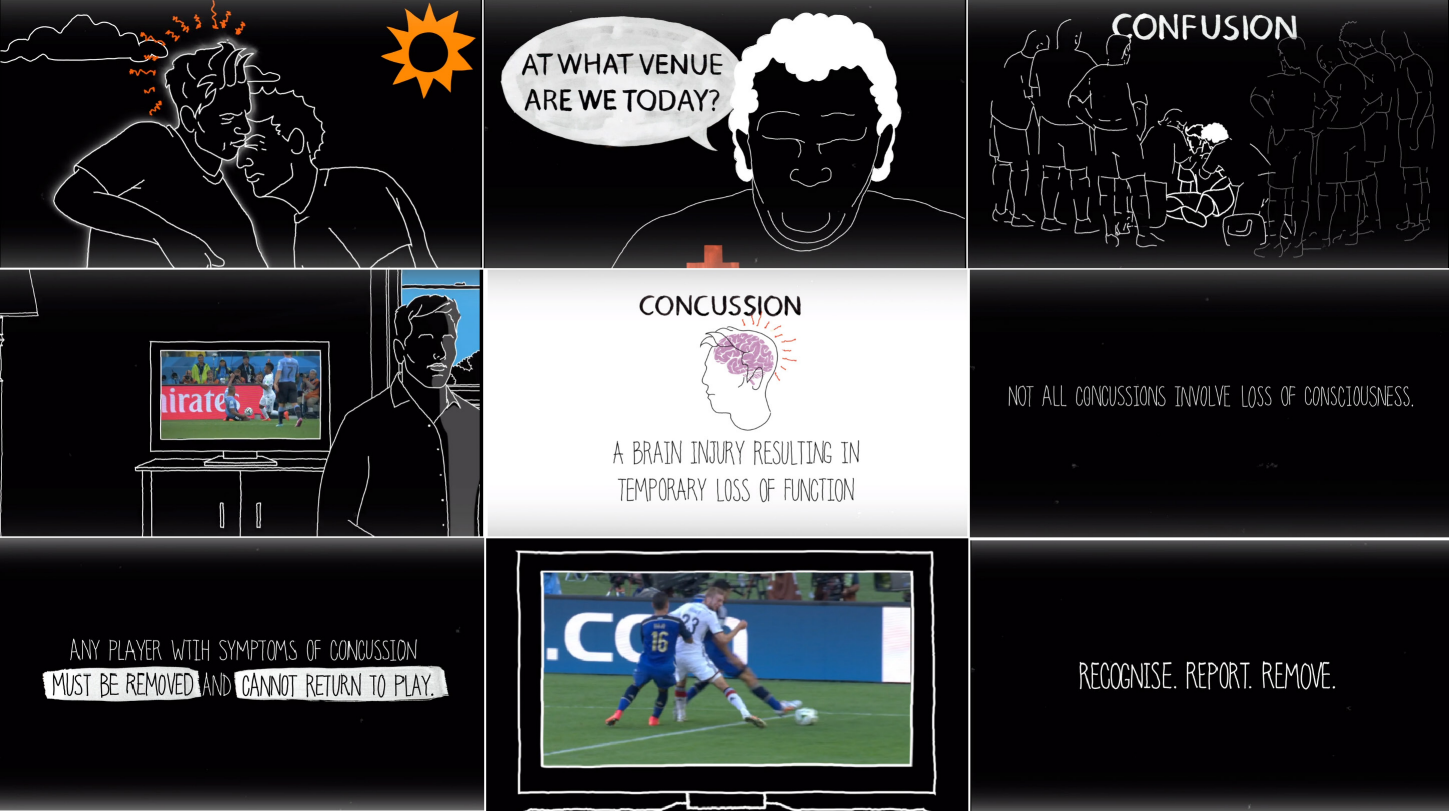
The educational concussion module for professional footballers.

All information gathered in the previous steps were synthetised and discussed with a creative partner selected for the development of the animated video. Video footage of three concussive events that occurred during the 2014 World Cup in Brazil were kindly provided by FIFA. An introductory video was recorded with two high-profile professional footballers, stating the importance of a valid management of concussion and the importance of the concussion module. An animated video lasting 2.5 min and using video footage of concussive events during World Cups was created. Screenshots of the animated video are presented in figure 1.

## Feasibility and effect of the concussion module

### Methods

A quasiexperimental study based on a one-group pretest post-test design was conducted.^13 14^ This study did not meet the criteria for the Medical Research Involving Human Subjects Act and therefore did not require approval from a Dutch human ethics research committee. This study was conducted in accordance with the Declaration of Helsinki and the Dutch Personal Data Protection Act. The participants consisted of professional footballers recruited by FIFPro. Inclusion criteria were: (1) current professional footballer; (2) 16 years or older; (3) able to read and comprehend texts in English. With regard to the nature of the study, a convenience sample of around 60 professional footballers was recruited.^14^

Knowledge about and attitude towards concussion was based on the Rosenbaum Concussion Knowledge and Attitudes Survey (RoCKAS) and its Concussion Knowledge Index (CKI) and Concussion Attitudes Index (CAI).^15^ The CKI consists of true/false questions and questions about the recognition of common concussion symptoms (with eight non-scored distractors).^15^ A total score ranging from 0 to 25 is calculated, with a higher score representing greater concussion knowledge.^15^ The CAI consists of Likert scale (1–5) questions, with the answer reflecting the safest practice receiving 5 points and the least safe practice receiving 1 point.^15^ A total score ranging from 15 to 75 is calculated, with a higher score representing safer concussion attitude.^15^ Feasibility of the concussion module was operationalised as (1) relevance of the concussion module, (2) added value of the concussion module, (3) form of the concussion module appropriate and (4) duration of the concussion module appropriate. These outcomes were measured on a 5-point scale (from ‘1: strongly disagree’ to ‘5: strongly agree’).

Two English electronic questionnaires (one pretest and one post-test) were set up, including all outcome measures related to knowledge and attitude (see online supplementary appendix 1). The outcome measures related to feasibility were embedded in the post-test questionnaire (see online supplementary appendix 1), while the following descriptive variables were added to the pretest questionnaire: age, height, body weight, duration of professional football career, field position and level of play. Each questionnaire took about 10 to 15 min to complete. Potential participants were informed about the study (aim and procedures) by FIFPro via email. Participants interested in the study gave their informed consent (electronically) and were given access to the pretest questionnaire, which they were asked to complete within 2 weeks. After completion of the pretest questionnaire, participants were given access to the concussion module and were required to view it within 2 weeks. Finally, participants received the post-test questionnaire, which they were asked to complete within 2 weeks. The responses to both questionnaires were anonymised for reasons of privacy and confidentiality. Once completed, the electronic questionnaires were saved automatically on a secured electronic server that only the principal researcher could access. All players participated voluntarily in the study and did not receive any reward for their participation.

10.1136/bmjsem-2018-000490.supp1 Supplementary data

All data analyses were performed using the statistical software IBM SPSS Statistics 25.0 for Mac. Descriptive data analyses (mean, SD, frequency, range) were performed with all variables from the pretest and post-test questionnaires. Potential increase in knowledge and attitude was explored by comparing the pretest and post-test scores of the RoCKAS with the non-parametric Wilcoxon signed-rank test (p<0.05).^13 16^

### Results

The results of the study are presented in table 1. A total of 61 professional footballers (78% male; 22% female) gave their informed consent and were enrolled in the study. Their mean age, height, weight and career duration was 24 years (SD=6), 177 cm (SD=10), 72 kg (SD=10) and 6 years on average (SD=5), respectively. Fifty-five per cent had not incurred a concussion during their football career, 33% reported one or two concussions, and 12% reported three or more concussions. The mean CKI score of the participants was 18.8 (SD=2.8) at pretest and 20.0 (SD=2.0) at post-test (not significant, Z=213; p=0.16).

**Table 1:** Effect of the educational concussion module: participants’ characteristics, concussion knowledge and attitude and view of professional footballers

		Characteristics (n=61)	
Gender (male/female; %)		78/22	
Age (in years; mean±SD)		23.8±5.7	
Height (in cm; mean±SD)		177.1±9.7	
Weight (in kg; mean±SD)		72.1±10.4	
Duration of football career (in years; mean±SD)		5.7±5.2	
History of concussion (%)			
None		55	
One or two		33	
Three or more		12	
	**Pre test**	**Post-test**	
Concussion knowledge (0–25; mean±SD)	18.8±2.8	20.0±2.0	P=0.16
Concussion attitude (15–75; mean±SD)	60.2±8.3	64.2±6.7	P=0.01
	**View of professional footballers**		
Concussion module is relevant (%)		97	
Concussion module of added value (%)		87	
Suitable duration of the concussion module (%)		100	
Suitable form of the concussion module (%)		100	

The mean CAI score of the participants was 60.2 (SD=8.3) at pretest and 64.2 (SD=6.7) -- a significant increase in concussion attitude (Z=331; p=0.01). Ninety-seven per cent of the participants rated the concussion module as relevant to professional footballers, and 87% mentioned that it had added value for their knowledge/attitude towards concussion. Both form and duration of the concussion module were positively assessed by all participants (100%).

## Discussion

Our quasiexperimental study (pretest post-test design) among 61 professional footballers revealed that the attitude (CAI) mean score increased after viewing the concussion module (only significant for CAI). The large majority of the participants was positive about the relevance and added value of the concussion module; they were unanimously satisfied with its duration and form.

### The educational concussion module for and by professional footballers

We developed the module using a structured and systematic processes often used in health-related research.^10–12^ Accordingly, the first step was to explore the needs and view of professional footballers towards an educational concussion module. Such a needs assessment is an essential step that should be required within the development of any intervention, allowing relevant groups (especially end-users) to contribute to the problem analysis and to be involved in defining the contours of a potential solution.^10–12^ The needs assessment offers the possibility to collaborate with end-users, which might ultimately increase the chances of wide uptake and implementation of the developed intervention.^17^ Professional footballers and their national unions were involved in the whole process of the development of our educational module, from the problem analysis to the definition of the contours of the module. Therefore, both qualitative (interviews) and quantitative (survey) approaches were applied among more than 300 players. These footballers expressed the need for an educational concussion module to enhance their knowledge about and attitude towards the injury.

### Concussion knowledge and attitude in professional football and other sports

Using the same questionnaire as in our study (RoCKAS), Williams *et al*^18^ explored concussion knowledge and attitude of 21 professional footballers from one English Football League Championship club (mean age: 23 years).^18^ The authors found a mean knowledge (CKI) score of 16.4 (SD=2.9) and a mean attitude (CAI) score of 59.6 (SD=8.5), which is lower than in our sample.^18^ Two other studies about concussion knowledge and attitude by means of the RoCKAS were recently conducted among collegiate and kickboxing athletes (18 years of age or older).^19 20^ In 430 collegiate athletes from a variety of sports, the mean knowledge (CKI) score was 19.7 (SD=2.2) and the mean attitude (CAI) score was 58.6 (SD 8.0), while those scores among 193 competitive kickboxing athletes were 19.5 (SD=2.3) and 62.7 (SD=7.4), respectively.^19 20^ By comparison to these two studies, it seems that the mean knowledge (CKI) and attitude (CAI) scores in the professional footballers enrolled in our study were similar, both before (pretest) and after (post-test). However, with regard to those scores and the maximal score that can be obtained for concussion knowledge (25) and attitude (75), professional footballers need to be educated thoroughly about concussion, for instance through the implementation of our educational concussion module.

### Implementation of the educational concussion module

According to the needs of professional footballers and their national unions, the educational concussion module should be implemented in professional football to empower the knowledge and attitude of players towards the injury. For such an implementation, several strategies might be applied, for instance through club visits conducted by national professional footballers’ unions during which players are informed and educated about diverse topics. Another strategy might be to make the education of players about concussion mandatory during their precompetition medical assessment prior every season. Such an approach was successful in the English professional rugby where a 100% completion rate is achieved among players, coaches and referees. With regard to their higher risk for concussions, female professional footballers should also be educated towards the injury and therefore, the female version of the educational concussion module can be used.

## Conclusion

The educational concussion module led to better attitude of professional footballers towards the injury. Players were unanimously positive about its relevance, added value, form and duration. We recommend the educational concussion module be implemented in professional football.
