# Supplementary material for: Educational concussion module for professional footballers: from systematic development to feasibility and effect
Source: BMJ Open Sport Exerc Med. 2019 Mar 7;5(1):e000490. doi: 10.1136/bmjsem-2018-000490 (PMC6407555; doi:10.1136/bmjsem-2018-000490)
Supplement: Supplementary data [file bmjsem-2018-000490supp001.docx]

Appendix 1. Items of the questionnaire

*Concussion Knowledge Index (CKI)*

1. There is a possible risk of death if a second concussion occurs before the first one has healed.

2. Running everyday does little to improve cardiovascular health.

3. People who have had one concussion are more likely to have another concussion.

4. Cleats help players’ feet grip the playing surface.

5. In order to be diagnosed with a concussion, you have to be knocked out.

6. A concussion can only occur if there is a direct hit to the head.

7. Being knocked unconscious always causes permanent damage to the brain.

8. Symptoms of a concussion can last for several weeks.

9. Sometimes a second concussion can help a person remember things that were forgotten after the first concussion.

10. Weightlifting helps to tone and/or build muscle.

11. After a concussion occurs, brain imaging (e.g., CAT Scan, MRI, X-Ray, etc.) typically shows visible physical damage (e.g., bruise, blood clot) to the brain.

12. If you receive one concussion and you have never had a concussion before, you will become less intelligent.

13. After 10 days, symptoms of a concussion are usually completely gone.

14. After a concussion, people can forget who they are and not recognize others but be perfect in every other way.

15. High-school students and college students tend to be the same age.

16. Concussions can sometimes lead to emotional disruptions.

17. A player who gets knocked out after getting a concussion is experiencing a coma.

18. There is rarely a risk to long-term health and well-being from multiple concussions.

19. Symptoms: Hives Feeling in a “Fog” Headache Weight Gain Difficulty Speaking Feeling Slowed Down Arthritis Reduced Breathing Rate Sensitivity to Light Excessive Studying Difficulty Remembering Difficulty Concentrating Panic Attacks Dizziness Drowsiness Hair Loss

20. Scenario: While playing in a game, Player Q and Player X collide with each other and each suffers a concussion. Player Q has never had a concussion in the past. Player X has had 4 concussions in the past.

It is likely that Player Q’s concussion will affect his long-term health and well-being.

It is likely that Player X’s concussion will affect his long-term health and well-being.

21. Scenario: Player F suffered a concussion in a game. She continued to play in the same game despite the fact that she continued to feel the effects of the concussion.

Even though Player F is still experiencing the effects of the concussion, her performance will be the same as it would be had she not suffered a concussion.

*Concussion Attitude Index (CAI)*

1. I would continue playing football while also having a headache that resulted from a minor concussion.

2. I feel that coaches need to be extremely cautious when determining whether a player should return to play.

3. I feel that mouthguards protect teeth from being damaged or knocked out.

4. I feel that professional players are more skilled at their sport than academy players.

5. I feel that concussions are less important than other injuries.

6. I feel that a player has a responsibility to return to a game even if it means playing while still experiencing symptoms of a concussion.

7. I feel that a player who is knocked unconscious should be taken to the emergency room.

8. I feel that most academy players will play professional football in the future.

9. Scenario: Player R suffers a concussion during a game. Coach A decides to keep Player R out of the game. Player R’s team loses the game.

I feel that Coach A made the right decision to keep Player R out of the game.

Most players would feel that Coach A made the right decision to keep Player R out of the game.

10. Scenario: Player M suffered a concussion during the first game of the season. Player O suffered a concussion of the same severity during the semi-final playoff game. Both players had persisting symptoms.

I feel that Player M should have returned to play during the first game of the season.

Most players would feel that Player M should have returned to play during the first game of the season.

I feel that Player O should have returned to play during the semi-final playoff game.

Most players feel that Player O should have returned to play during the semi-final playoff game.

11. Scenario: Player R suffered a concussion. Player R’s team has a physical therapist/doctor on the staff.

I feel that the physical therapist/doctor, rather than Player R, should make the decision about returning Player R to play.

Most players would feel that the physical therapist/doctor, rather than Player R, should make the decision about returning Player R to play.

12. Scenario: Player H suffered a concussion and he has a game in two hours. He is still experiencing symptoms of concussion. However, Player H knows that if he tells his coach about the symptoms, his coach will keep him out of the game.

I feel that Player H should tell his coach about the symptoms.

Most players would feel that Player H should tell his coach about the symptoms.

*Feasibility*

1. The concussion module is relevant for professional footballers.

2. The concussion module has added value for my knowledge/attitude towards concussion.

3. The form of the concussion module (animated video with video footages) is appropriate.

4. The duration of the concussion module (+/- 3min 30s) is appropriate.
